# A cross-sectional survey assessing the acceptability and feasibility of self-report electronic data collection about health risks from patients attending an Aboriginal Community Controlled Health Service

**DOI:** 10.1186/1472-6947-14-34

**Published:** 2014-04-16

**Authors:** Natasha E Noble, Christine L Paul, Mariko L Carey, Robert W Sanson-Fisher, Stephen V Blunden, Jessica M Stewart, Katherine M Conigrave

**Affiliations:** 1 Priority Research Centre for Health Behaviour, School of Medicine and Public Health, The University of Newcastle, Callaghan, NSW, Australia; 2 Casino Aboriginal Medical Service, Casino, NSW, Australia; 3 National Health Performance Authority, Level 9, Oxford St, Sydney NSW 2001, Australia; 4 Sydney Medical School, University of Sydney, Sydney, NSW, Australia

**Keywords:** Australia, Aboriginal, Touch screen questionnaire, Health risk factors, Screening

## Abstract

**Background:**

Aboriginal Australians experience significantly worse health and a higher burden of chronic disease than non-Aboriginal Australians. Electronic self-report data collection is a systematic means of collecting data about health risk factors which could help to overcome screening barriers and assist in the provision of preventive health care. Yet this approach has not been tested in an Aboriginal health care setting. Therefore, the aim of this study was to examine the acceptability and feasibility of a health risk questionnaire administered on a touch screen laptop computer for patients attending an Aboriginal Community Controlled Health Service (ACCHS).

**Methods:**

In 2012, consecutive adult patients attending an ACCHS in rural New South Wales, Australia, were asked to complete a health risk survey on a touch screen computer. Health risk factors assessed in the questionnaire included smoking status, body mass index, and level of physical activity. The questionnaire included visual cues to improve accuracy and minimise literacy barriers and was completed while participants were waiting for their appointment.

**Results:**

A total of 188 participants completed the questionnaire, with a consent rate of 71%. The mean time taken to complete the questionnaire was less than 12 minutes. Over 90% of participants agreed that: the questionnaire instructions were easy to follow; the touch screen computer was easy to use; they had enough privacy; the questions were easy to understand; they felt comfortable answering all the questions.

**Conclusions:**

Results indicate that the use of a touch screen questionnaire to collect information from patients about health risk factors affecting Aboriginal Australians is feasible and acceptable in the ACCHS setting. This approach has potential to improve identification and management of at-risk individuals, therein providing significant opportunities to reduce the burden of disease among Aboriginal Australians.

## Background

As for other Indigenous populations worldwide [1], Aboriginal and Torres Strait Islander Australians experience significantly poorer health and younger mortality than other Australians [2,3]. The high prevalence of modifiable risk factors, such as smoking and high body mass, contributes significantly to this health gap [3-8]. Cancer screening rates are also known to be low [9,10]. Therefore, the potential to reduce the disease burden experienced by Aboriginal and Torres Strait Islander Australians by targeting change in these common risk and screening behaviours is considerable [11].

Aboriginal Community Controlled Health Services (ACCHSs) are services designed to provide culturally appropriate health care to Aboriginal communities [12]. Approximately 50% of the Aboriginal population of Australia access ACCHSs for their health care [13], making ACCHSs an appropriate setting in which to address the risk status of Aboriginal Australians.

The provision of preventive care requires the identification of at-risk patients [14]. However, reliance on medical records to identify at-risk patients may be limited by the accuracy and completeness of such records [14-16]. Significant gaps in the recording of risk factor status in Aboriginal and Torres Strait Islander health settings have been identified [14,16,17]. For example, across more than 60 Indigenous community health centres, weight and BMI were not recorded for an average of 45% and 78% of patients respectively, while only 28% of patients were identified as smokers, relative to known higher smoking rates in sampled communities [17]. Such inconsistencies in detecting or recording data for at-risk patients may be due to the time required for screening, and privacy or sensitivity concerns [17,18]. For example, many Aboriginal health workers are themselves smokers, and may feel uncomfortable assessing the smoking status of their patients and providing cessation advice [19,20]. The need to prioritise across a complex array of health and other problems facing many Aboriginal patients is a further challenge to achieving regular assessment of modifiable health risks and the provision of preventive health care [14,21].

Electronic data collection utilising portable devices offers a potentially effective means of collecting self-reported risk factor information from patients. Electronic data collection is preferred by patients [22,23], reduces missing values compared to paper and pencil methods [24], allows tailoring of questions to minimise patient burden, and has the potential for automatic entry of data into medical records [23,25].

The acceptability of computer based approaches to health data collection has been explored in a limited sample of indigenous and/or socially disadvantaged populations worldwide. For example, an audio touch screen computer-assisted self-interviewing questionnaire to collect lifestyle risk data was well accepted by a cohort of American Indians; although lower education levels and infrequent computer use in the past year were predictors of useability problems [26]. A touch screen kiosk providing smoking cessation information was also successful in engaging a sample of low-literacy, underserved, Mexican-American primary care patients [27].

Studies specifically in the Australian Aboriginal health care setting are uncommon. One study utilised a laptop questionnaire to collect risk and resilience data [23], and another touch activated hand held computer devices to collect data about condom use [28], from Aboriginal youth. However neither study reported survey consent or completion rates. Hunter and colleagues reported on the acceptability of a touch-screen kiosk in two Indigenous-specific settings (a health clinic and a Centrelink office serving a discrete Aboriginal community) which provided visual, audio and printed information about musculoskeletal disorders and diabetes [29]. Of note was the importance of including non text-based information and culturally appropriate graphics to promote acceptability in this population [29].

Electronic data collection therefore holds potential as an efficient and systematic means of collecting data about health risk factors to assist in the provision of preventive health care for Aboriginal people. An electronic approach to health risk data collection would overcome some of the key barriers for health service staff such as limited time and sensitivity restraints. However, it has not been tested for this purpose in an Aboriginal health setting. The aims of this study were therefore to examine the acceptability and feasibility of an electronic health risk questionnaire administered on a touch screen laptop computer for adult patients attending an ACCHS. Feasibility and acceptability were assessed in terms of: i) patient consent rates and consent bias; ii) survey completion rates; iii) time taken to complete the questionnaire; iv) the proportion of patients needing assistance to complete the questionnaire; and v) patient feedback on the survey.

## Methods

### Study design and setting

A health risk questionnaire was administered using a touch screen laptop to patients attending an ACCHS in regional New South Wales (NSW). The acceptability data presented here were part of a larger cross-sectional study about the prevalence of a range of health risk factors and demographics. Questionnaires were completed anonymously to minimise response bias and encourage participant consent. Data collection occurred over approximately nine weeks in early 2012. Ethics approval for the project was granted by the Human Research Ethics Committees of the University of Newcastle and the Aboriginal Health and Medical Research Council of NSW.

### Questionnaire development

**Figure 1:**
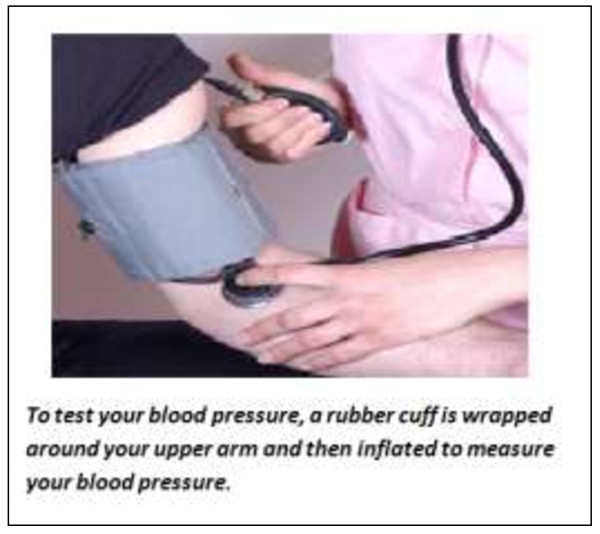
Example of a visual description included in the touch screen questionnaire to minimise literacy barriers.

The questionnaire was designed with a year seven school reading age, and included pictures and limited text in order to minimise reading demands. For example, a visual illustration was presented when asking patients about timing of their last blood pressure test (Figure 1); a standard drinks chart, plus the number of standard drinks in common bulk packages of alcohol was shown to assist in answering questions about alcohol consumption. Input into the design of the questionnaire and survey recruitment methods was sought from collaborators experienced in Aboriginal health research and during pilot testing with Aboriginal ACCHS staff and patients.

### Participants

All patients attending the ACCHS for a general practice (GP) appointment, aged 18 years or older, not too sick to participate and able to provide informed consent were eligible. All patients including Aboriginal and non-Aboriginal patients were invited to participate and all participants gave informed consent.

### Procedure

Patients were approached by a Research Assistant (RA) and invited to complete the touch screen questionnaire in a quiet section of the waiting room while waiting for their appointment. A non-Aboriginal RA was present to set up and oversee survey administration. An Aboriginal RA assisted with approaching patients for approximately half of the recruitment period (due to logistical recruitment problems and low patient throughput on some days). Participants were able to exit the questionnaire if called in for their appointment prior to completion. Results for interrupted questionnaires were included if at least 75% of the questionnaire was completed (with unanswered responses recorded as missing). A RA offered assistance with questionnaire completion as required, and noted whether participants received assistance (none/some assistance/interview style). A RA also recorded the estimated age and gender of non-consenting patients in order to assess consent bias.

### Measures

The touch screen questionnaire included demographic questions (age, gender, Indigenous and marital status, highest level of education, source of income, overcrowding and exposure to physical or emotional violence) and items assessing the health risk status of participants (body mass index, smoking status, alcohol consumption, level of physical activity, consumption of fruit and vegetables, alcohol intake, drug use, depression and adherence with screening guidelines). Measures were either previously validated or drawn, where possible, from Indigenous specific surveys such as the National Aboriginal and Torres Strait Islander Health Survey and Social Survey [30-32]. The questionnaire also included five acceptability statements; participants were asked to indicate whether: the instructions were easy to follow; the touch screen was easy to use; they had enough privacy; the questions were easy to understand; they felt comfortable answering all the questions (yes/no/not sure). Participants were also asked whether they would be willing to complete a similar survey at future appointments. The survey was modified for the final 32 participants in order to identify whether for future surveys, participants would be happy for their doctor to see a copy of their questionnaire responses.

### Materials

Digivey Survey Suite software (CREOSO Digivey Survey Centre, Arizona, USA) was used to design and administer the touch screen questionnaire. The RA demonstrated how to navigate through the questionnaire using the ‘next’ or ‘back’ buttons. Participants could skip questions by touching the ‘next’ button. Branching algorithms were used to tailor questions to individual participants (for example, only females over 50 years of age were asked about breast cancer screening).

### Statistical analyses

Chi-square analysis was used to compare the age groups, gender and Aboriginal status of consenting and non-consenting participants. Aboriginal status of consenting participants was only recorded for participants who completed the questionnaire, and not for non-consenters or those who exited the questionnaire before completion; therefore the Aboriginal status of the final complete sample was compared to the proportion of total active Aboriginal and non-Aboriginal patients usually attending the ACCHS. Aggregate data on the Aboriginal status of all adult patients who had attended the Service at least three times in the last two years was extracted from ACCHS medical records for comparison.

Parametric tests (*t-*tests and one-way ANOVA) were used to compare the time taken to complete the questionnaire for Aboriginal and non-Aboriginal participants and across age groups. Chi-square and Fisher’s exact test (where cells had small expected frequencies) were used to compare the acceptability of the questionnaire, assistance required to complete the questionnaire, and willingness to complete a similar survey in the future, for Aboriginal status and across age groups.

## Results

i. Consent rates and consent bias

**Figure 2:**
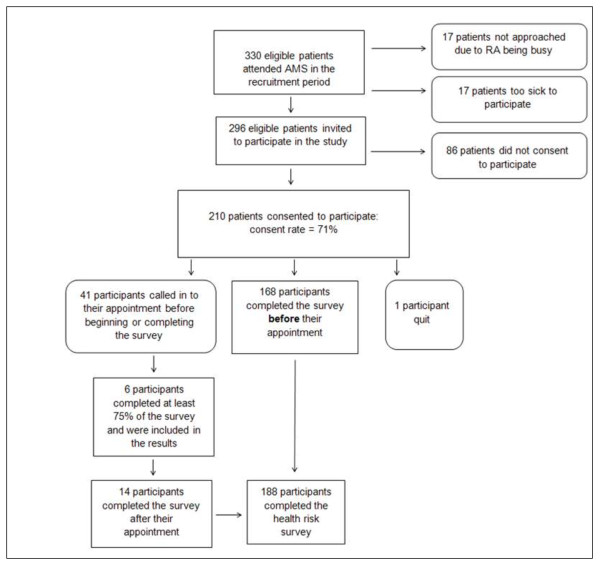
Flow diagram of participant recruitment.

Approximately 330 eligible patients attended the ACCHS during the recruitment period. Figure 2 illustrates the number of participants at each stage of recruitment and survey completion. A total of 296 eligible patients were approached and 210 consented to complete the questionnaire, giving a consent rate of 71%. There was no difference in consent rate associated with the different RAs.

The gender and approximate age groups of consenting and non-consenting participants are shown in Table 1. There were no significant differences in the gender (*χ*^2^(1, *N* = 296) = 1.87, *p* = .17) or age (*χ*^2^ (5, *N* = 296) = 2.13, *p* = .83) of consenting and non-consenting patients. However, non-Aboriginal patients were significantly overrepresented in the final sample compared to the proportion of non-Aboriginal patients usually attending the ACCHS, *χ*^2^ (1, *N* = 1437) = 18.69, *p* < 0.01.

ii. Completion rates and missing data

**Table 1:** Demographics of consenting study participants and non-consenting patients

	**Consenting patients (n = 210)**	**Non-consenting patients (n = 86)**	**Total patients (consenting and non-consenting)**	**Medical record data**	***p*-value**
	**n (%)**	**n (%)**			
**Gender**					0.17
Male	82 (67%)	41 (33%)	123 (100%)	-	
Female	128 (74%)	45 (26%)	173 (100%)	-	
**Age**					0.61
<35 yrs	76 (73%)	28 (27%)	104 (100%)	-	
35-54 yrs	82 (72%)	32 (28%)	114 (100%)	-	
≥55 yrs	52 (67%)	26 (33%)	78 (100%)	-	
**Indigenous status**^**a**^					<0.01
Aboriginal^b^	135	-	n/a	1056	
Non-Aboriginal	53	-	n/a	193	

^a^Indigenous status was only recorded for 188 participants who completed the survey. The Indigenous status of participants who consented but did not complete the survey was not recorded, and the Indigenous status of non-consenters was also not recorded.

^b^The sample included 1 participant who identified as Torres Strait Islander and 3 participants who identified as both Aboriginal and Torres Strait Islander. For simplicity, the term ‘Aboriginal’ is used to refer to all participants who identified as being of either Aboriginal and/or Torres Strait Islander origin.

Of the 210 participants who consented to the survey, a total of 168 participants (80%) completed the questionnaire before attending their appointment (see Figure 2). Complete data were available for all these participants, except for missing depression data for one participant who chose to skip all depression screening questions, and three participants who accidently skipped one of the nine depression screening items.

iii. Time taken to complete the questionnaire

Questionnaire duration for those who completed the questionnaire before their appointment (*n* = 168) could only be extracted for 165 participants due to a technical problem. The mean time taken to complete the questionnaire was 11 mins 8 secs (*SD* = 4 min 56 sec), and 85% of participants completed the questionnaire in less than 16 mins. Aboriginal participants (*n* = 117) took significantly more time to complete the questionnaire (*M* = 11 mins 43 sec, *SD* = 5 min 5 sec) than non-Aboriginal participants (*n* = 48; *M* = 9 mins 41 sec, *SD* = 4 min 15 sec), *t*(163) = 2.44, *p* = .016. Older participants also took longer to complete the questionnaire than younger participants (*F*(5, 159) = 7.62, *p* < .01). For example, the mean time taken by participants aged under 25 (*n* = 29) was 9 mins 10 sec (*SD* = 3 min 51 sec), while for those aged over 65 (*n* = 13) the mean time was 16 mins 30 sec (*SD* = 6 min 47 sec).

iv. Assistance sought/accepted in completing the questionnaire

Of the 206 participants who began the questionnaire (see Figure 2), 75% (*n* = 154) completed the questionnaire without assistance, 19% accepted some assistance (*n* = 39), and 6% completed the questionnaire in an interview style (*n* = 13). The proportion of participants who sought or accepted assistance with completing the questionnaire, across Aboriginal status and age groups, is shown in Table 2.

**Table 2:** Proportion of participants seeking/accepting assistance with the questionnaire by Aboriginal status and age group

	**No assistance n (%)**	**Some assistance n (%)**	**Interview style n (%)**	***p*-value**
**Indigenous status**^**a**^				0.5
Aboriginal^b^ (*n* = 135)	98 (73%)	27 (20%)	10 (7%)	
Non-Aboriginal (*n* = 53)	43 (81%)	8 (15%)	2 (4%)	
**Age group**				<.01
<35 yrs (*n* = 73)	64 (88%)	9 (12%)	0	
35-54 yrs (*n* = 82)	63 (77%)	14 (17%)	5 (6%)	
≥55 yrs (*n* = 51)	27 (53%)	16 (31%)	8 (16%)	

^a^Indigenous status was only available for participants who completed the questionnaire (*n* = 188).

^b^The sample included 1 participant who identified as Torres Strait Islander and 3 participants who identified as both Aboriginal and Torres Strait Islander. For simplicity, the term ‘Aboriginal’ is used to refer to all participants who identified as being of either Aboriginal and/or Torres Strait Islander origin.

Assistance with questionnaire completion did not differ significantly between Aboriginal and non-Aboriginal participants (Fisher’s exact test *p* = .5), but did differ between age groups (Fisher’s exact test *p* < .01). After grouping participants into three age groups, almost half of those participants aged 55 years and over sought or accepted assistance with the questionnaire (either some assistance or interview: 47%), compared to 12% of those aged under 35 yrs and 23% of those aged 35–55 years.

v. Participant feedback on the acceptability of the touch screen questionnaire

**Figure 3:**
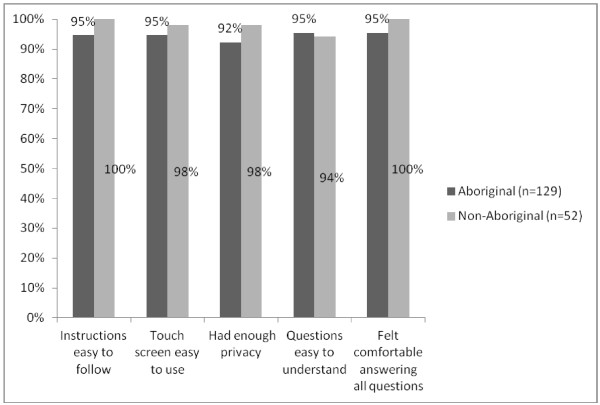
Percentage of Aboriginal and non-Aboriginal participants who agreed with each of the questionnaire acceptability items.

Questionnaire acceptability questions were completed by 181 participants. The proportion of participants who responded positively (‘yes’ response) to these items, according to Aboriginal status, are shown in Figure 3. There were no significant differences in agreement with any of the statements between Aboriginal and non-Aboriginal participants, or across age groups (for those aged under 35 yrs, 35-55 yrs and over 55 yrs; Fisher’s exact test: all *p*s > .05).

Ninety-six percent of participants indicated that they would be willing to complete a similar questionnaire at future ACCHS visits, either ‘sometimes’ (44%), ‘most of the time’ (19%) or ‘every time’ (33%). Willingness to complete a future questionnaire did not differ significantly between Aboriginal and non-Aboriginal participants (Fisher’s exact test *p* = .90) or across age groups (between those aged under 35 yrs, 35-55 yrs and over 55 yrs; Fisher’s exact test *p* = .94). Finally, of the subsample of participants (*n* = 32) who were asked whether, for future questionnaires, they would be willing for their doctor to see a copy of their questionnaire answers, 91% (*n* = 29) indicated ‘yes’.

## Discussion

Study results indicate that a large majority of patients who attended the ACCHS were willing and able to complete the touch screen health risk questionnaire. The consent rate was high (71%), and did not appear to differ according to age or gender, although Aboriginal patients were slightly less likely to consent; missing data was minimal. The majority of patients (80%) were able to complete the questionnaire in the time that they were waiting to see the doctor, and most patients (75%), independent of their Aboriginal status, were able to complete the questionnaire without any assistance, although older participants were more likely to need some help. Results suggest that an electronic screening approach such as the one used here could be readily incorporated in routine clinical practice during appointment waiting times, requiring minimal staff time for gathering risk factor information, provided sufficient staffing resources were available to encourage patients to complete the questionnaire and assist if needed. The potential clinical utility of this approach is further supported by the high degree of acceptability of the questionnaire, and the high proportion of patients who indicated that they would be willing to complete a similar questionnaire at future appointments (96%), and for their doctor to see their results for future questionnaires (91%). Perhaps most encouraging was the high proportion of participants who agreed that they felt comfortable answering all the questions, despite the inclusion of questions about potentially sensitive issues such as smoking, alcohol consumption and illicit drug use.

Several study limitations should be noted. The questionnaire was completed anonymously, and therefore consent rates and acceptability levels may not reflect patients’ responses had the questionnaire results been linked to their medical record or otherwise available to their doctor or health worker. Clearly this is an important consideration if such a system were to be implemented in routine clinical practice. The other major limitation relates to the validity of self-reported health risk factor information. Although validated measures were used wherever possible [33-35], many show only moderate sensitivity and specificity (such as short measures of physical activity and diet) and the validity of most measures for use in the Aboriginal population has not been specifically established. While validation was outside the scope of this study, an exploration of the appropriateness and accuracy of such measures would be an important consideration for the clinical utility of the questionnaire results. Additional study limitations relate to the generalisability of the results given that the study was conducted in a single ACCHS in a rural but non-remote setting. In more remote communities, literacy issues or less computer experience may impact on questionnaire acceptability and utility. However, the use of a touch screen with graphics and multimedia options such as those used in audio computer-assisted self-interviewing methods [36], or the multimedia Queensland ‘HITnet’ program for Indigenous communities [37,38] could help to overcome these barriers.

This study confirms that the use of a touch screen laptop survey is both feasible and acceptable for the systematic collection of risk factor status and screening adherence among adult patients attending an ACCHS. The observed consent rate, acceptability of the questionnaire, and willingness to complete similar future questionnaires, suggest that this approach is a potentially sustainable one which could be implemented more widely as part of routine practice in the ACCHS setting, subject to a comprehensive evaluation of the validity of the health risk data collected. If results were linked directly into patient medical records, this would assist health care providers with the systematic identification of at-risk patients, reduce the burden on providers associated with risk assessment, and leave more time available for the provision of preventive care. Provided that collected data are reliable, questionnaire results could provide a starting point for providers to initiate discussion about potentially sensitive issues such as smoking, alcohol or drug use. Screening is also likely to raise personal awareness among patients about their health risks and screening needs.

## Conclusions

This study has provided evidence for the potential acceptability and usefulness of an electronic health risk assessment tool for use with patients of ACCHSs. Ultimately, this kind of systematic approach to risk factor assessment has the potential to improve the identification and therefore the management of at-risk individuals, therein providing a significant and much needed opportunity to reduce the burden of disease among Aboriginal Australians.

## Competing interests

The authors have no competing interests to declare.

## Authors’ contributions

NN developed the study materials, conducted data collection, undertook analysis and interpretation of the data, and drafted the manuscript. CP made substantial contributions to the study conception and design, provided support during data collection, and assisted with drafting and revision of the manuscript. MC made substantial contributions to the study conception and design, and provided critical feedback and revision of the manuscript. RSF made substantial contributions to the study design, facilitated data collection and revised the manuscript. SB provided feedback on study design and specific study materials, facilitated data collection, and revised the manuscript. JS and KC provided input into the study design, materials and specific survey items, and provided critical feedback and revision of the manuscript. All authors gave final approval of the version to be published.

## Pre-publication history

The pre-publication history for this paper can be accessed here:

http://www.biomedcentral.com/1472-6947/14/34/prepub
